# Early clinical exposure in medical education: the experience from Debre Tabor University

**DOI:** 10.1186/s12909-023-04221-4

**Published:** 2023-04-17

**Authors:** Biniam Ewnte, Tegbar Yigzaw

**Affiliations:** 1 Debre Tabor University, Gondar, Ethiopia; 2 Jhpiego Ethiopia, Addis Ababa, Ethiopia

**Keywords:** Early clinical exposure, Undergraduate Medical Education, Vertical integration

## Abstract

**Background:**

The idea of early clinical exposure in Ethiopian medical schools is a young concept. Old and newly established universities across the nation are shifting towards incorporation of early clinical exposure (ECE) in their curricula. Debre Tabor University introduced ECE in undergraduate medical education from inception. This study generated evidence on students’ experience and academic leaders’ reflection on early clinical exposure implementation.

**Objective:**

This study was carried out to investigate medical students’ perception towards early clinical exposure and its implementation process by instructors in undergraduate medical education at Debre Tabor University.

**Method:**

A cross-sectional survey design that combines quantitative and qualitative methods was conducted in 2021. We asked fifth year medical students (42) to complete a self-administered questionnaire on 5-point Likert scale. The data were supplemented by semi-structured interview with 6 purposively selected academic leaders on the factors that facilitate or impede early clinical exposure implementation. The quantitative data were entered and analyzed using SPSS 20 to compute frequency, median and interquartile range. The qualitative data were analyzed thematically.

**Results:**

The study findings suggest that early clinical exposure (ECE) has a positive impact on the development of students’ professional knowledge, problem-solving skills, motivation, active learning, and community orientation. Specifically, 64.3% of the surveyed students believed that ECE was effective in constructing their professional knowledge, while 52.4% felt that it improved their problem-solving skills and facilitated constructive/active learning. Additionally, 57.1% of students reported that ECE improved their motivation and 50% noted that it facilitated community orientation. The study also identified several barriers to the implementation of ECE, with the heavy workload being the most commonly mentioned (78.6%). Other challenges included a loose linkage between academic and healthcare institutions (59.5%) and a lack of orientation on the implementation process (35.7%). Academic leaders reflected that ECE was beneficial in familiarizing students with the clinical environment, but staff commitment was crucial for its successful implementation. The study also found that heavy workload, lack of assessment dedicated to ECE on the curriculum, and poorly oriented staff about the program impeded its implementation.

**Conclusion and recommendations:**

The findings of this study suggest that early clinical exposure was beneficial learning method. Teachers’ commitment to their roles with adequate preparation, and the contribution of curriculum in providing the learning objective and cases for each session were factors that facilitate effective implementation of ECE. Heavy workload and poor orientation about the program could impede ECE implementation.

## Background

The first medical school in Ethiopia was established by the Addis Ababa University School of Medicine using a Conventional Curriculum, in which basic science courses were provided in the first two years, followed by clinical training in the third year in a totally independent manner. The basic sciences courses were not only isolated from the clinical sciences courses, but they were also taught in a discipline-based manner without any horizontal integration [1, 2].

The current Ethiopian harmonized national medical curricula were built on the strengths of the conventional one; with incorporation of innovative teaching strategies like problem-based learning (PBL), vertical integration through early clinical exposure, horizontal integration of biomedical courses while social and population health courses are delivered in a parallel fashion. This curriculum is currently being implemented across all medical schools of the country. It lasts for a total duration of 6 years.

This curriculum was developed in part to fill the gaps found in the demands of general practitioners who were actually practicing. The evaluation revealed that clinical and internship years were the most helpful to their future medical practice and that more practical skill training is required in medical education. They underlined the significance of enhancing the therapeutic relevance and focus of basic sciences [3, 4].

In a vertically integrated curriculum, early experience with clinical problems and in clinical settings is interspersed with continued science teaching, be it less and less over time. In contrast, in a conventional curriculum, classroom teaching is programmed in the first years of medical school, and clinical training in the final years [5].

Debre Tabor University (DTU) medical school introduced “hybrid innovative curriculum” adapted to the Ethiopian context. The curriculum is competency-based curriculum building on strengths of the traditional curriculum and incorporating innovative and transformative features highlighted in the SPICES model (student-centered, problem-based, integration, community-based, systematic) and other global recommendations on instructional design [6, 7].

As opposed to the practice in the conventional curriculum, in Debre Tabor University undergraduate medicine curriculum, clinical exposure starts in year one with progressive increase in intensity, complexity and student responsibility over the years [6]. It is a compulsory part of the curriculum. It’s held twice weekly, each session lasting two hours in a clinical skills lab setting, hospital, or household. The exposure occurs under clinician supervision.

The clinical exposure session of each week consists of case related to the week’s topic in a system-based module. Students arranged in groups of ten each have clinical skills laboratory every week on Thursday following lectures and PBL sessions of the week. The clinical skills learning features role play, practice with clinical simulators (high and low fidelity), and video demonstrations.

Following the clinical skills lab session, there is a practical session conducted in the teaching hospital, health center, home visits, and community setting. The task for each week is adapted to the objectives of the particular session.

Teaching in a clinical setting is characterized as training that is patient- and problem-centered and generally involved directly with patients. Increasing number of students, competing needs between teaching students versus treating patients, opportunistic approach, and fewer number of patients could make clinical teaching challenging(Spencer, 2003) [8].

Early clinical exposure (ECE) is a teaching-learning approach that encourages medical students to interact with patients as early as their first year of medical school. This will increase the case exposure of students across their study years, overcoming opportunistic approach of traditional clinical teaching giving learners more satisfaction [9].

**Fig. 1:**
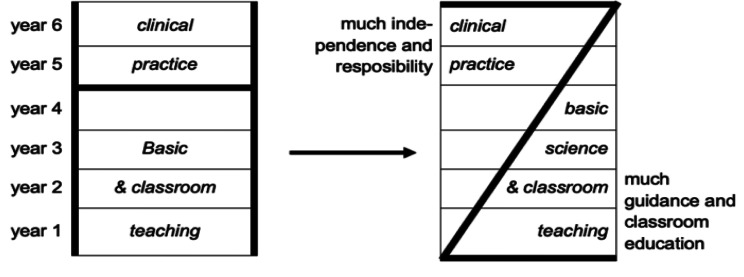
The traditional H-shaped medical curriculum and a Z-shaped curriculum model [5]

Various educational systems are embracing the concept of an integrated curriculum, in which clinical contact occurs in the early years and basic science instruction goes beyond the usual first two years [8]. The provision of patient contact in the early years of the curriculum, in both clinical and community settings was felt to be particularly important. These activities motivated students, provided experiences on which to reflect and promoted integration of learning [10, 11]. Vertical integration merges learning and practice, which does not stop at licensing or certification [5] Vertical integration in medical education may be viewed as a reorganized curriculum structure (as a move from an H to a Z shape) (see Fig. 1). In vertical integration, the traditional H-shaped medical curriculum is replaced by a Z-shaped curriculum model (adopted from [5] with permission).

The early years of the curriculum provide students with valuable introduction to the physician professional role in clinical practice, experiences that mimic their future roles, opportunities for reflection, and rehearsal of the skills involved in managing these experiences [12, 13]. Students were found to value opportunities to become acclimated to clinical settings before they are thrust into clerkships [14]. Students also reported increased self-esteem and enhanced enthusiasm for the study of medicine as a result of early patient contact by observing outpatient consultations [15].

Early clinical exposure was also found to improve student satisfaction with the first two years’ experience and provides a structure for teaching patient-centered, integrated clinical medicine, which is important in the general professional education of all students [16]. Students who passed through early clinical exposure teaching were found to become respectful of a patient’s life and illness experiences, and their understanding of communication as central to a doctor’s daily work increased [17].

Students were found to clearly enjoy the experience of early clinical exposure and perceived that it was valuable when it was used as an alternative approach to reinforce didactic instruction in basic science teaching, it was also recommended that it is adaptable to other basic science topics subjects as well (19, 25).

In a PBL context, vertical integration of basic sciences and clinical medicine has been demonstrated to drive profound rather than superficial learning, resulting in a better comprehension of important biomedical principles. Integration probably leads to better retention of knowledge and the ability to apply basic science principles in the appropriate clinical context [18].

The preclinical patient contacts were instrumental in giving students sense of well preparedness for the clerkships: they did not experience a major gap between the preclinical and clinical phase [19]. Students with clinical exposure were significantly more satisfied with their medical education than were their peers who lacked such exposure [20]. The importance in helping for lifelong learning has also been noted [21].

However, implementation of early clinical exposure is not without challenges. Lack of many opportunities on real patients, unenthusiastic tutors to teach in junior years, ill-defined learning objectives and confusion about how much depth in which they were expected to learn the skills are identified as challenges in early clinical exposure amongst students [22]. The need for deep involvement of teachers and their enthusiasm has been emphasized for successful implementation of vertical integration [18].

The idea of early clinical exposure in Ethiopian medical schools is a young concept. Old universities as well as newly established ones across the nation are shifting towards incorporation of early clinical exposure in their curricula. This study generated evidence on the student’s experience of such practice, as the learner experience has a pivotal role in any teaching learning activity. In addition, the study narrated academic leaders’ reflection on early clinical exposure. This study has explored the practice of early clinical exposure in a setting which can be classified as low resource. Which will try to add to the growing knowledge and practice of early clinical exposure in such medical schools.

To the best knowledge of the authors, no study was conducted in Ethiopia to evaluate the implementation and assess advantages or possible drawbacks of early clinical exposure in medical education. This study has explored the overall picture of early clinical exposure among medical students and academic leaders at a glans. It will support future studies in the field have a base to start with.

## Methods

This study was carried out to investigate medical students’ and academic leaders’ perception of early clinical exposure implementation in undergraduate medical education at Debre Tabor University.

Early clinical exposure refers to the integration of clinical experiences into the pre-clinical years of medical education, which allows students to gain practical experience and develop clinical reasoning skills before entering clinical clerkships.

The study utilized a mixed-methods design, which involved both quantitative and qualitative data collection methods to obtain comprehensive insights into the perceptions of medical students and academic leaders. The study population consisted of all 5th year medical students (Quantitative part) and academic leaders (Qualitative part) at Debre Tabor University. A total of 358 medical students and 22 academic leaders were included in the study.

The quantitative data were collected through a structured questionnaire, which was distributed to all medical students. The questionnaire included questions about their perceptions of early clinical exposure, its benefits and challenges, the adequacy of resources available for implementation, and suggestions for improving the implementation of early clinical exposure. The qualitative data were collected through focus group discussions and in-depth interviews with academic leaders.

The data collected from the study were analyzed using both descriptive and inferential statistics for quantitative data, while qualitative data were analyzed thematically.

## Results

A total of 42 fifth year medical students returned a completed questionnaire, which makes the response rate 100%. The mean age of students was 23.8 ± 1.0 years.

Six academic leaders (2 deans, 1 clinical coordinator, 1 EDC coordinator and 2 department heads), who had at least 1 year experience implementing the curriculum participated in the qualitative interviews.

Students’ perception of the actual benefits gained due to early clinical exposure was assessed in terms of five main factors. These include the contribution of ECE for constructing professional knowledge (three items), developing problem-solving skills (three items), improving motivation to learn (three items), encouraging constructive/active learning (three items) and exposing for community orientation (three items). Table 1 provide the results of students’ level of agreement, with median and inter quartile range for each of the items and main factors.

**Table 1:** Number and percentage of students

Description	Frequency	Percent
Female	6	14.3
Male	36	85.7
Total	42	100.0

### Construction of professional knowledge

Most respondents (N = 27, 64.3%) reported agreement or strong agreement on the contribution of ECE in construction of professional knowledge (M = 4, IQR = 1.0) (Table 1). Students perceived that this happened mainly through improving students subject matter understanding (N = 31, 73.8%), while most of them agree ECE has contribution to understand underlying mechanisms (M = 4, IQR = 2.0) (Table 2).

**Table 2:** Students’ perception of ECE as an educational strategy among 5th year medical students, Debre Tabor University, 2014 (N = 42)

S.no	Item			SDA n(%)	DA n(%)		N n(%)			A n(%)		SA n(%)		M (IQR)	
**Construction of Professional knowledge**				**4(9.7)**	**4(9.7)**		**7(16.6)**			**19(45.2)**		**8(19.0)**		**4.00 (1.00)**	
1	Improve subject matter understanding			2 (4.8)	2 (4.8)		7(16.7)			22(52.4)		9(21.4)		4.00(1)	
2	Integrate knowledge across disciplines			4 (9.5)	4 (9.5)		9 (21.4)			19 (45.2)		6(14.3)		4.00(1)	
3	Helps to understand underlying mechanisms			7(16.7)	7(16.7)		5(11.9)			14(33.3)		9(21.4)		4.00(2)	
**Problem solving skills**				**7(16.6)**	**6(14.3)**		**7(16.6)**			**19(45.2)**		**3(7.1)**		**4.00 (2.00)**	
1	To solve real-world problems			10 (23.8)	6 (14.3)		8(19.0)			16(38.1)		2(4.8)		3.00(2.25)	
2	To consider alternatives when solving a problem			7 (16.7)	6 (14.3)		7 (16.7)			21(50.0)		1(2.4)		4.00(2)	
3	To make reasonable conclusions			5(11.9)	5(11.9)		7(16.7)			21(50.0)		4(9.5)		4.00(1.25)	
**Motivation**				**3 (7.1)**	**4 (9.5)**		**11 (26.2)**			**18 (42.8)**		**6 (14.3)**		**4.00 (1.00)**	
1	Motivate interest for learning			4 (9.5)	3 (7.1)		10(23.8)			18(42.9)		7(16.7)		4.00(1)	
2	Enhanced professional identity			2 (4.8)	6 (14.3)		10 (23.8)			20(47.6)		4(9.5)		4.00(1)	
3	Motivate to serve underserved population groups			3 (7.1)	3 (7.1)		12 (28.6)			16 (38.1)		8(19.0)		4.00(1)	
**Active learning**				**7(16.6)**	**8 (19.0)**		**5 (11.9)**			**17 (40.5)**		**5 (11.9)**		**4.00 (2.00)**	
1	Encourages to express your own opinions			7(16.7)	10(23.)		4(9.5)			16(38.1)		5(11.9)		3.50(2)	
2	Encourages to react positively to feedback and criticism			7 (16.7)	8 (19.0)		6(14.3)			16(38.1)		5(11.9)		3.50(2)	
3	Encourages to provide a reason behind your opinions			6(14.3)	6(14.3)		7(16.7)			19(45.2)		4(9.5)		4.00(2)	
**Community orientation**				**9(21.4)**	**5 (11.9)**		**7 (16.6)**			**10 (23.8)**		**11 (26.2)**		**3.50 (3.00)**	
1	To better understand the culture and custom of the community			10(23.)	6(14.3)		5(11.9)			9(21.4)		12(28.6)		3.50(3.25)	
2	To experience a broader view on the health needs of the community			10 (23.8)	4(9.5)		8(19.0)			10(23.8)		10(23.8)		3.00(2.5)	
3	To understand health problems outside a hospital setting			8(19.0)	6(14.3)		8(19.0)			10(23.8)		10(23.8)		3.00(2.25)	

SDA = Strongly disagree, DA = Disagree, N = Neutral, A = Agree, SA = Strongly agree, M = Median, IQR = Inter-quantile range

### Problem solving skills

Almost half of the respondents (N = 22, 52.4%) reported agreement or strong agreement with a statement asking “ECE develops their problem-solving skills” (M = 4, IQR = 2.0), particularly in considering alternatives when solving a problem (N = 23, 54.7%), and making reasonable conclusions (N = 25, 59.5%) (Table 1). However, students’ perception on the contribution of ECE to solve real-world problems was dispersed among response options (M = 3, IQR = 2.25) (Table 2).

### Motivation

Many students (N = 24, 57.1%) agreed or strongly agreed with regards to the improvement of motivation by ECE (M = 4, IQR = 1.0), by motivating students’ interest for learning (N = 25, 59.5%), enhancing their professional identity (N = 24, 57.1%), and making them to serve underserved population groups (N = 24, 57.1%).

### Active learning

Table 1 shows that a little more than half of the students (N = 22, 52.4%) agreed on the contribution of ECE in creating constructive/active learning (M = 4, IQR = 2.0) and similar response was found on encouraging them to express their own opinions and to react positively to feedback and criticism (N = 21, 50%).

### Community orientation

Half of the students’ (N = 21, 50.0%) stated their agreement or strong agreement on the role of ECE in providing students a community orientation (M = 3.5, IQR = 3.0). However, students’ response on the role of ECE in supporting them to understand the culture and custom of the community (M = 3.5, IQR = 3.25), to experience a broader view on the health needs of the community (M = 3.0, IQR = 2.5), and to understand the frequency and type of health problems outside a hospital setting (M = 3.0, IQR = 2.25) was dispersed among response options (Table 2).

Most students agree ECE has played significant role in terms of motivation, making them active learners and acquisition of problem-solving skills (Table 3).

**Table 3:** Summary of students’ perception of ECE as an educational strategy among 5th year medical students, Debre Tabor University, 2014 (N = 42)

Variables	Mean (SD)	Median	IQR		
			25 Percentiles	50 Percentiles	75 Percentiles
Professional knowledge	3.5 (1.19)	4.0	3.0	4.0	4.0
Problem solving skills	3.1 (1.25)	4.0	2.0	4.0	4.0
Motivation	3.4 (1.08)	4.0	3.0	4.0	4.0
Active learning	3.1 (1.32)	4.0	2.0	4.0	4.0
Community orientation	3.2 (1.51)	3.5	2.0	3.5	5.0

SD = standard deviation, IQR = interquartile range

#### Perceived barriers of ECE implementation

Table 4 provides student perceptions of barriers to ECE implementation in DTU. The top three barriers to the successful implementation of ECE were heavy workload (78.6%), poor academic and health institutions linkages (59.5%), and lack of orientation on the process of implementation (35.7%).

**Table 4:** Perceived Barriers of ECE Implementation among final year medical students, DTU (N = 42)

s.no	Item	Not Barrier N (%)	Barrier N (%)	Rank
1	Lack of orientation on the process of implementation	27(64.3)	15(35.7)	3
2	Poor teaching skill from tutors	30 (71.4)	12 (28.6)	4
3	Lack of motivation from teachers/ tutors	31(73.8)	11 (26.2)	5
4	Heavy workload	9 (21.4)	33 (78.6)	1
5	Poor academic and health institutions linkages	17 (40.1)	25 (59.5)	2

#### Qualitative data result on ECE implementation

Six academic leaders who were directly involved in the implementation of ECE participated (Table 5).

**Table 5:** Academic positions, qualifications and experience of academic leaders

Academic position	Qualification	Years of Experience
Dean	MD, Surgeon	4
Dean	MD, Surgeon	2
Clinical coordinator	General practitioner	2
EDC coordinator	Masters in Anesthesia	3
School Head	MD, Surgeon	1
School Head	MD, Surgeon	1

Five main themes were discovered from the data sources. The first theme focuses on the benefit of ECE, the second and third deals with the factors that facilitate and impede ECE implementation, respectively. The fourth and fifth themes were all about institutional and programmatic issues related to ECE implementation, respectively.

### Theme-1 effectiveness of ECE in facilitating students’ learning

Respondents indicated that ECE was effective in facilitating student learning by showing their future career and understanding the role of basic science which arouses their interest in learning. This is supported by the following extract:

*‘It familiarizes them with patients, prepares them to be competent in clinical skills, especially during starting clinical years, so that they will not be confused or disoriented when they encounter patients; and allows them to quickly meet the desired skills and objectives while initiating clinical years.‘ Clinical coordinator*.

### Theme-2 factors that facilitate ECE implementation

Respondents identified the main factor that affects ECE implementation was teachers’ commitment to their roles, particularly their adequate preparation for all sessions which in turn leads to improved group learning habit, punctuality as well as professionalism. The following extract from data yielded the subthemes of teachers’ related factor that facilitate effective implementation of ECE:

*‘It is well recognized that teachers are willing and dedicated to collaborate, provide constructive criticism, and be sufficiently prepared in a learning objective for each session.‘ School head*.

### Theme-3 factors that impede ECE implementation

This theme had three subthemes: factors from the side of students, academic staff, and the curriculum that impede ECE implementation.

#### Students

In the first session, there is confusion among students and sometimes they feel fatigued related to the heavy workload imposed by other learning activities. The following extract from data yielded the subthemes of student’s related factors that impede the effective implementation of ECE.

*‘Because since day one class one; there is class, PBL, skills lab. They came to the hospital visit after the skills lab session. They get exhausted and get bored easily. They practice skills with models at skills lab first it’s after that they get to the hospital and visit real patients.’ Dean*.

#### Staff

Hospital physicians, who do not hold academic position with the University, get confused about students’ level of learning and the objectives of each session when they coach students. Therefore, physicians from hospital need to be oriented about the curriculum and early clinical exposure activities.

*‘Physicians from the hospital engaged in coaching students haven’t taken any orientation about the curriculum, so they often go deeper in teaching students. School head*.

#### Curriculum

The assessment method is not defined clearly and the checklists are not aligned with session objectives. Students clerk patients but their clinical skill is not measured and incorporated as part of summative assessment. The following quote from data yielded the subthemes of curriculum related issues that impede effective implementation of ECE.

*‘Related to curriculum, gaps identified where there is no clearly stated summative assessment method and there is no dedicated percentage mark for early clinical exposure evaluation.’ EDC coordinator*.

### Theme-4 institutional issues

This theme had three subthemes: Infrastructure, the need for more instructors and institutions relationship.

#### Infrastructure

Respondents agreed that ECE by its nature demands resources. Although schedules for clinical practice are prepared ahead of time, delay and shortage of transport service to clinical practicum sites remains a major hindrance to implementation. One extract from data under the subtheme of ‘infrastructure for ECE implementation’ was given below.

*‘Finding****transportation****for clinical and community practicum teaching is a significant difficulty for us.’ Clinical coordinator*.

#### Need for more instructors

In a clinical practice session there may be 50–60 students and four or five clinical teachers to supervise them. Because of the high clinical teacher to student ratio, all students may not have equal chance of exposure and physicians from the hospital side may be involved in teaching activities with no orientation on the objectives of sessions and the curriculum. This could result in inappropriate matching of student competence level with preceptor expectations. Example of extract from data under the subtheme of ‘the need for more instructors is as follows.

*‘Keeping track of their (students’) numbers can be tough. Because the majority of practicum sessions are done in internal medicine wards, it might be difficult to keep track of their numbers.‘ School Head*.

#### Institutional relationship

The academic leaders indicated that there is a signed memorandum of understanding between the teaching institution and clinical site to provide clinical practice opportunities for students. However, they claimed that relationship between the hospital and the university was getting worse due to the conflict of interest related to payment among hospital physicians working with a joint appointment.

*‘There is a payment problem.‘ When a student is paired with a preceptor who has a conflict of interest, the preceptor may not be able to offer the degree of mentorship or clinical teaching that is required.’ Dean*.

### Theme-5 programmatic issues

This theme had two subthemes: Organization and coordination and the existence of ECE implementation monitoring mechanism.

#### Organization and coordination

The coordination starts smoothly within the department for which the clinical teaching focal person is assigned who is mainly responsible for preparing and revising schedules. At the college level, the clinical coordinator is responsible for preparing checklists according to the objective of particular system. Both university and hospital physicians are involved in the teaching and are assigned by the clinical coordinator. Clinical teachers teach according to set objectives. For example, if it is respiratory system, students will see patients with respiratory problems. Teachers will select cases in advance. Under the supervision of teachers and using the checklist they are provided, students take brief history, perform physical examination and suggest differential diagnosis. Teachers will assist if students face any challenges while clerking patients. Following clerking, students will present what they have worked on. The papers will be collected and submitted to the respective department head, to be used only for formative assessment purpose even though the department heads don’t give feedback to students based on the papers. The subtheme ‘Organization and coordination’ was derived by the following examples from the data.

*‘… The clinical coordinator is in charge of the early clinical exposure activities as a whole. Students visit the hospital, clerk, and present, but this is used for grading purpose. There is no specific percentage for evaluating early clinical exposure.‘ Clinical coordinator*.

#### Monitoring mechanism

The educational development center of the college along with the clinical coordinator oversees the implementation of early clinical exposure. The EDC evaluates the quality of checklists used at practicum site and monitors alignment of selected cases with the objectives of the session. The subtheme ‘Monitoring mechanism’ was derived from the following examples from the data.

*‘As part of assuring quality, we regularly evaluate the quality of the checklists used in ECE and also check the alignment between the cases of the week and the set objectives’ EDC coordinator*.

## Discussion

The purpose of this study was to investigate experiences and perceptions of early clinical exposure implementation in undergraduate medical education in Debre Tabor University. The majority of medical students thought that ECE could help them study more effectively in the preclinical phase. Students perceived that ECE benefited them in constructing professional knowledge, improving problem-solving skills, increasing motivation to learn, creating active learning and promoting community orientation.

The majority of students in this study considered ECE was effective in construction of professional knowledge as it provides opportunity to understand underlying mechanisms, enhance in-depth understanding of subject matter and integrate knowledge across disciplines which in turn enables to appreciate the application of basic sciences knowledge in clinical problem. These views agree with findings of studies conducted in Iran [23] and India [24, 25] which reported that ECE had positive influence in knowledge and skills gained by students. Early clinical experience provides medical students with an experiential context for learning basic science content and opportunities to experience the relevance between basic science knowledge and its clinical application. This enables medical students to obtain a better and deeper understanding of medicinal theory and practice through the application of their knowledge in real hospital situations [26] .

This study revealed that the great majority of the students perceived that ECE improved their problem-solving skills, as it increased their ability to solve real- problems of critically ill patients, providing reasonable conclusions and encouraged them to consider alternatives when solving patients’ problems. Other studies [24, 27] have also reported that ECE enhances the logical reasoning skills of the students. Instead of studying by rote, they would learn to think rationally. They will benefit from the development of an analytical mind as they progress through their professional life.

Also, the results of the current study demonstrated that ECE helps students to develop fundamental clinical skills as well as a moral attitude with active learning. Students reported that ECE program increased students’ interest and motivation for learning and facilitated active learning. These findings seem to be consistent with those of other researchers, who reported early contact with patients can increase medical students’ enthusiasm and motivation in their education [13, 23, 25, 28]. Interacting with other clinical students, clinical faculty, physicians, and patients seemed to have stimulated them and allowed them to discuss and share their knowledge. Furthermore, it’s possible that familiarity with the physicians’ expected duties and responsibilities helped these students. Evidences also show that interactions with and between peers and faculty are important in professional development [23, 29] .

Students perceived that heavy work load, poor academic and health institutions linkages and lack of orientation on the process of implementation were barriers for ECE implementation. These views are in agreement with other studies [13, 24] which reported more time should be devoted to the conduct of ECE sessions and huge content was covered in a short time, some of the content was irrelevant and unnecessary.

### Limitation of the study

As this is a cross sectional study based on the self-evaluation of students, it is prone to subjectivity since the students may not be sincere and over-evaluate their own performance. All aspects of ECE might not have been covered by the tool and method used. Instructors directly engaged in conducting ECE were not surveyed in this study. This could affect the input that could have been found from their perspective.

## Conclusions

This study adds to the growing body of evidence that ECE was beneficial learning method as it was effective in construction of student’s professional knowledge, improving their problem-solving skills, motivating students’ interest for learning, creating constructive/active learning and orienting the community they are going to serve. Students also perceived that heavy work load, poor linkages/relationships between academic and health institutions, and lack of orientation on the process of implementation were barriers for ECE implementation.

The academic leaders revealed that ECE is an important education strategy and effective in facilitating students learning by showing their future career and understanding the role of basic science. Teachers’ commitment to their roles with adequate preparation, and the contribution of curriculum in providing the learning objective and cases for each session were factors that facilitate effective implementation of ECE.

Inadequate orientation for non-academic staff physicians, absence of summative assessment, shortage of transport service, and worsening hospital and university relationship were major factors that impede effective implementation of ECE. Regarding programmatic issues; clinical coordinators and department heads have had a better coordination for ECE implementation.

Therefore, all the above results provide evidence to support the introduction of ECE as a one teaching strategy by Debre Tabor University, College of Health Sciences. These findings show that implementation of ECE can be improved better by addressing the identified critical factors that hinder ECE implementation.

Future studies should ascertain improved attainment of clinical learning outcomes in an ECE curriculum using quasi-experimental study design.

## Data Availability

The datasets used and/or analyzed during the current study are available from the corresponding author on reasonable request.

## Abbreviations

DTU: Debre Tabor University
ECE: Early Clinical Exposure
FMoE: Federal Ministry of Education
FMoH: Federal Ministry of Health
NIMEI: New Innovative Medical Education Initiative
PBL: Problem Based Learning
SD: Standard Deviation
